# Robustness of cortical and subcortical processing in the presence of natural masking sounds

**DOI:** 10.1038/s41598-018-25241-x

**Published:** 2018-05-01

**Authors:** M. Jerome Beetz, Francisco García-Rosales, Manfred Kössl, Julio C. Hechavarría

**Affiliations:** 1 Institute for Cell Biology and Neuroscience, Goethe-University, 60438 Frankfurt/M., Germany; 2 Present Address: Department of Behavioral Physiology and Sociobiology, Biozentrum, University of Würzburg, Am Hubland, Würzburg, 97074 Germany

## Abstract

Processing of ethologically relevant stimuli could be interfered by non-relevant stimuli. Animals have behavioral adaptations to reduce signal interference. It is largely unexplored whether the behavioral adaptations facilitate neuronal processing of relevant stimuli. Here, we characterize behavioral adaptations in the presence of biotic noise in the echolocating bat *Carollia perspicillata* and we show that the behavioral adaptations could facilitate neuronal processing of biosonar information. According to the echolocation behavior, bats need to extract their own signals in the presence of vocalizations from conspecifics. With playback experiments, we demonstrate that *C. perspicillata* increases the sensory acquisition rate by emitting groups of echolocation calls when flying in noisy environments. Our neurophysiological results from the auditory midbrain and cortex show that the high sensory acquisition rate does not vastly increase neuronal suppression and that the response to an echolocation sequence is partially preserved in the presence of biosonar signals from conspecifics.

## Introduction

For understanding how nervous systems work, it is necessary to analyze neuronal processing of natural and ethologically relevant stimuli^1,2^. Since animals usually encounter a mixture of different stimuli, neuronal processing of ethologically relevant stimuli could be masked by the presence of irrelevant stimuli. Throughout this manuscript, we will refer to the relevant stimuli as “targets” and the irrelevant stimuli as “maskers”. Although, many behavioral adaptations, from here on called jamming avoidance responses (JARs) have been reported to reduce or even avoid masking^3–9^, it remains largely unexplored if neuronal processing of targets profit from the JARs. The present study reports JARs in the frugivorous bat *Carollia perspicillata* and tested if the JARs could facilitate neuronal processing of echolocation sequences in the presence of masking sounds.

Bats orientate acoustically by emitting biosonar calls that are reflected in surrounding objects^10–12^. For computing the distance to objects, bats measure the time interval from call emission to echo arrival, also called echo delay. The echo delay decreases linearly with decreasing distances. Since bats need to integrate call and echo information, the computation of distances could be interfered by acoustic maskers from call onset to echo arrival^13^. Neurons involved in distance processing, also called delay tuned neurons, integrate call and echo information to respond to certain echo delays^14–20^. Thus, bats represent good animal models to test if JARs could facilitate neuronal processing of distance information. Neurophysiologically, we focused on the inferior colliculus (IC) and the auditory cortex (AC) because most data from delay tuned neurons were obtained by recording neuronal signals from these two brain regions.

Our behavioral results show that *C. perspicillata* adapts its call emission pattern in the presence of maskers. The bats increase the tendency of emitting grouped calls and they reduce the call intervals (CIs) within call groups. High stimulus rates usually evoke neuronal suppression^21–23^ which has also been shown in *C. perspicillata*^24–26^. Thus, we tested if the increase in stimulus rate may also increase neuronal suppression which could impede target detection at the neuronal level. Our results show that in the IC, the neuronal suppression did not vastly increase and the neuronal tuning to the target was barely affected by maskers. In contrast, the suppression increased in the AC. But, target detection was higher in the AC than in the IC due to a higher neuronal selectivity in the AC. In summary, our results indicate that the JARs, reported here for *C. perspicillata*, could facilitate neuronal processing of the targets in the presence of maskers.

## Results

### Behavioral adaptations to acoustic masking during echolocation

**Figure 1:**
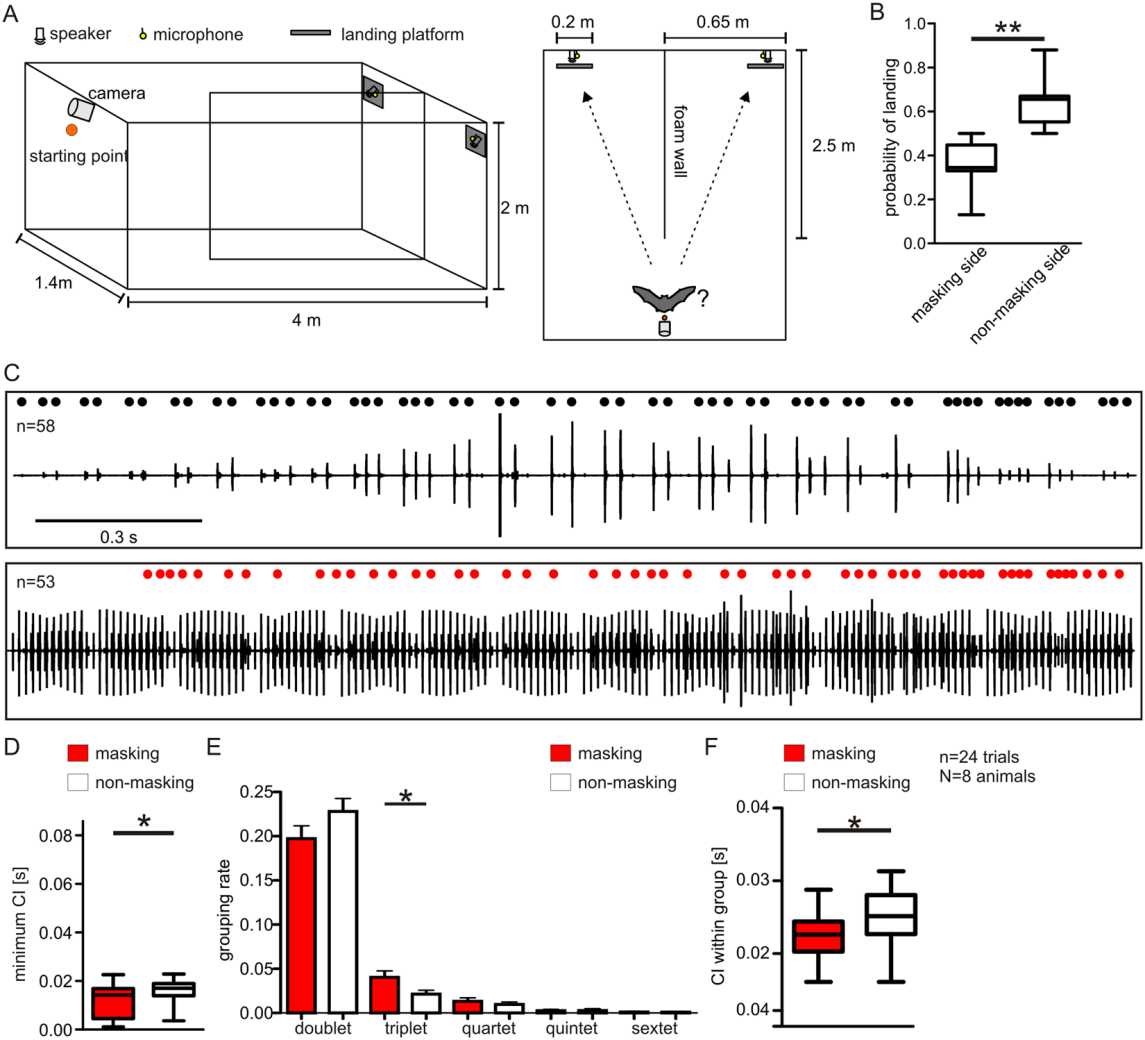
Behavioral adaptations of *C. perspicillata* when echolocating in noisy areas. (**A**) Schematic lateral (left) and top view (right) of the flight room. (**B**) Boxplots demonstrate the rate of landing on the platform at each side. (**C**) Representative oscillograms illustrate the call patterns of one bat in the absence (top; non-masking condition) and presence (bottom; masking condition) of maskers. Respectively, black and red dots represent time points of call emission during non-masking and masking conditions. *n* indicates the amount of emitted calls. Note that the lower oscillogram contains the masker in addition to the emitted calls. (**D**) Minimum call intervals (CIs) during masking and non-masking conditions. (**E**) Histogram shows the relative amount of call groups containing two (doublet), three (triplet), four (quartet), five (quintet) and six (sextet) calls in the non-masking and masking condition. (**F**) Boxplots illustrate the CIs within call groups.

In a flight room (Fig. 1A), the echolocation behavior from eight bats was monitored in the absence (training trials) and presence (test trials) of maskers. A wall, made out of foam, separated the room into two acoustically isolated sides. In training trials, under non-masking conditions, the hand released bats had to land on one out of two platforms at the end of the room (Movie S1). Two ultrasound sensitive microphones, each positioned behind a platform, recorded the call emissions. In test trials, under masking conditions, a masker was presented from one side of the room, whereas the contralateral side was silent. The masker represents a sequence of echolocation calls that was recorded during the first test trial from the tested bat (for details see methods). The bats preferred to land on the platform of the non-masking side (Paired t-test: p = 0.01; Fig. 1B; Movie S2). But, they could still echolocate and land on the platform at the masking side (Movie S3).

When comparing the call patterns emitted at the masking side during test trials with the ones emitted during training trials (exemplarily shown in Fig. 1C), it becomes evident that the bats changed their emission pattern in the test trials. Minimum CIs decreased during test trials (Wilcoxon signed rank test: p = 0.041, Fig. 1D). Median CIs, maximum CIs, and the number of emitted calls did not differ between the two conditions (Wilcoxon signed rank test: p = 0.432 for median CIs, p = 0.83 for maximum CIs, and p = 0.795 for number of calls; Fig. S1A,B). Behavioral studies reported that bats often emit groups of echolocation calls (*C. perspicillata*^27,28^, *Phylostomus discolor*^29^, *Eptesicus fuscus*^30–37^, *Myotis lucifugus*^38^, *Tadarida brasiliensis*^39^, and *Noctilio albivenris*^40^), a behavior that was also observed in the present study (signaled by black and red dots on top of each call pattern in Fig. 1C). During test trials, the bats did not change the relative amount of grouped calls (Fig. S1C; median = 59.7% under masking and 60.6% under non-masking; Paired t-test: p = 0.79). However, the bats increased the size of the call groups, indicated by the number of calls per call group (Figs 1E and S1D). The bats emitted significantly more triplets under masking than under non-masking conditions (Wilcoxon signed rank test: p = 0.046). Trials in which quartets, quintets, and sextets were emitted were too few for statistical comparison. Though, note that the mean values of the grouping rates for quartets and sextets were higher, under masking than under non-masking conditions (quartet = 10^−4^ vs. 13^−3^, sextet = 9^−4^ vs. 10^−4^, for non-masking vs. masking). Additionally, the bats decreased the median CI within the call groups from 25 to 22 ms (paired t-test: p = 0.02; Fig. 1F) which results into acoustic rates of 90 Hz (when considering call and echoes).

### Masker effect on single-unit responses in the inferior colliculus and auditory cortex

Acoustic rates higher than 40 Hz usually evoke neuronal suppression which is also the case for the IC^25^ and AC^26,41^ of *C. perspicillata*. Thus, the bat may only profit from the previously described JAR if the neuronal response to the target is not suppressed in the presence of the masker. The target was an echolocation sequence that mimicked a stimulus scenario in which the bat flies towards an object. Robustness of neuronal tuning to the target was tested by mixing the target with two different maskers (for details see methods) which leads to “mixture conditions”. The “single animal mixture” simulates a situation where two bats fly in the same room. “The colony mixture” simulates a situation in which many bats fly together. Note that the single animal masker condition resembles the situation that bats had to encounter in the behavioral experiments.

**Figure 2:**
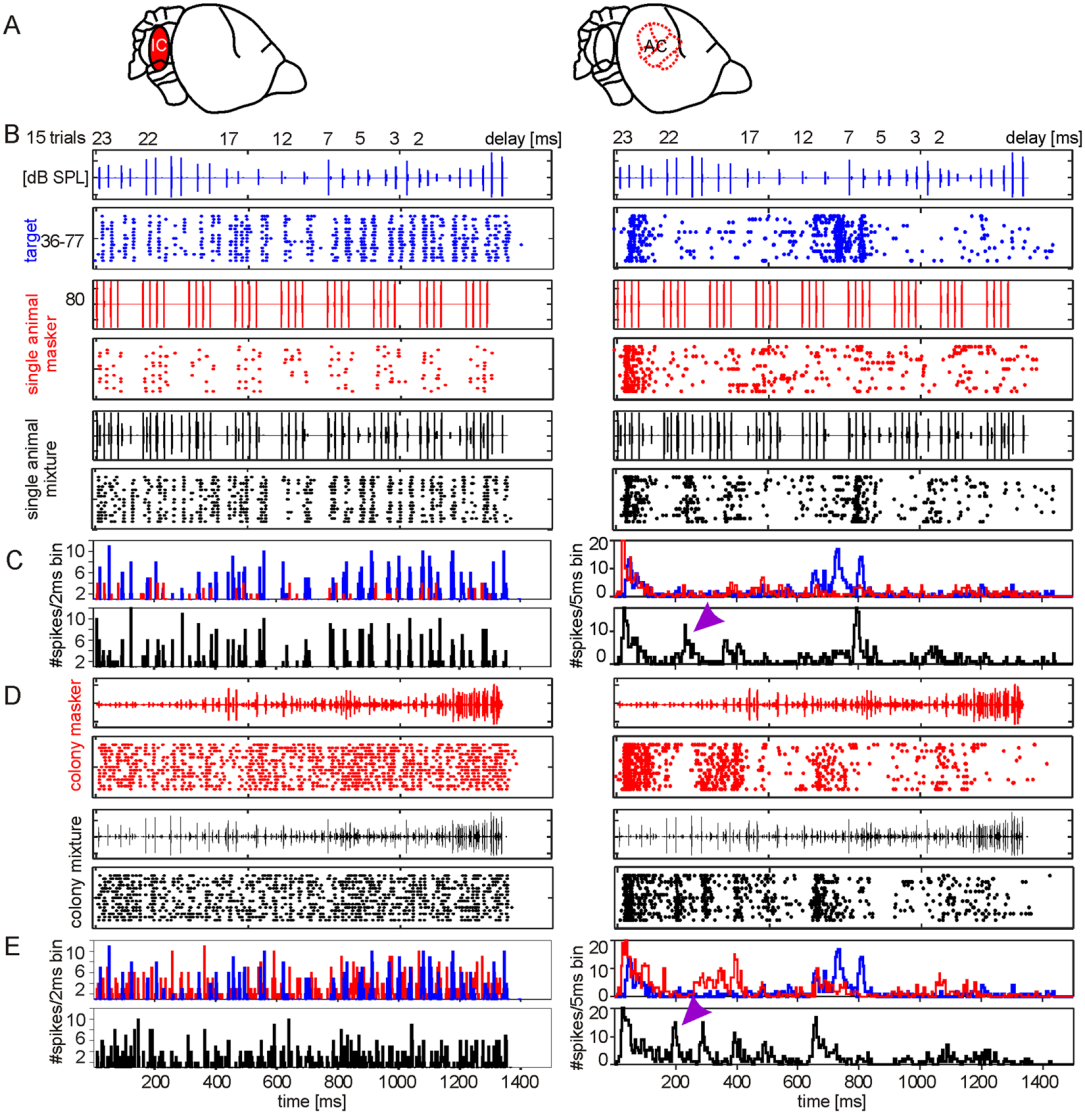
Neuronal responses of a collicular (left column) and a cortical (right column) unit. (**A**) Schematic lateral view on *C. perspicillata*’s brain. Recorded brain areas are highlighted (inferior colliculus (IC); auditory cortex (AC)). Raster plots (**B**,**D**) and PSTHs (**C**,**E**; binsize = 2 ms for collicular and 5 ms for cortical unit) show the neuronal activity of a representative collicular and cortical unit in response to the target (blue raster plot in A, blue PSTHs in **B** and **C**), to the single animal masker (red raster plot in **B**, red PSTH in **C**), to the colony masker (red raster plot in D, red PSTH in E), to the single animal mixture (black raster plot in B, black PSTH in **C**), and to the colony mixture (black raster plot in **D**, black PSTH in **E**). Oscillograms of the stimuli are indicated above each raster plot. Violet arrowheads point to neuronal responses elicited by a stimulus interference of the target and masker.

Respectively, neuronal activity from 49 collicular and 72 cortical single-units from six and nine bats was recorded. In response to the target, cortical units responded selectively to particular call-echo elements (Fig. 2B, right column). The latter stands in contrast to the collicular units (see example in Fig. 2B, left column) which responded to almost each acoustic event in the target echolocation sequence (see also Fig. S2). Both collicular and cortical units responded sparsely to the single animal masker (red raster plot and PSTH in Fig. 2B,C). The cortical unit showed a strong onset response which is common in the AC and is independent of the echo delay^26^. Therefore, the initial 150 ms of the cortical responses were excluded from the analysis. The neuronal firing pattern in response to the single animal mixture (black raster plots and PSTHs in Fig. 2B,C) was more similar to the response to the target than to the single animal masker. The latter was quantified by correlation values between the corresponding PSTHs (Wilcoxon signed rank test: p < 10^−5^; black crosses in Fig. S4A and B). In response to the single animal mixture, the cortical unit was selectively tuned to an echo delay of 7 ms (black raster and PSTH in the right column of Fig. 2B,C). In addition, between 200–400 ms after stimulus onset (violet arrowhead in Fig. 2C), a neuronal response was detected that was neither present in response to the target nor to the masker. This facilitation is caused by a stimulus integration of the target and the masker and may represent a neuronal correlate of jamming. Collicular and cortical neurons responded stronger to the colony than to the single animal masker (red raster plot and PSTH in Fig. 2). However, note that the cortical neurons responded more selectively to certain segments of the masker than the collicular neurons. The interference from the colony masker was higher than the one resulting from the single animal masker. The latter is indicated by higher similarities between the responses to the colony mixture and the colony masker than the ones between the colony mixture and the target (Paired t-test: p < 10^−5^; green crosses in Fig. S4A and B). The response pattern to the target was only poorly detectable in the colony mixture response (Fig. 2E).

**Figure 3:**
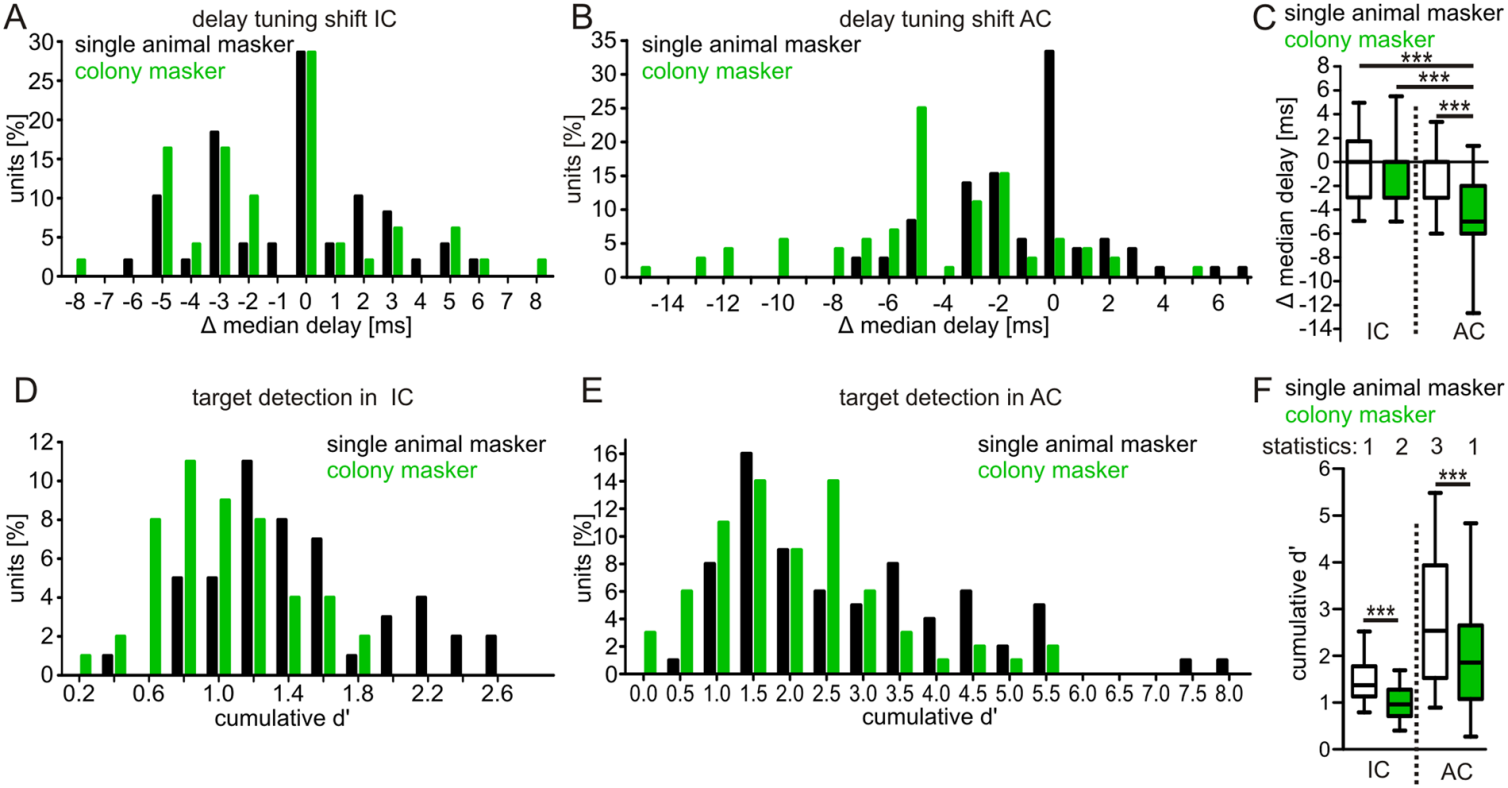
Robustness of neuronal processing in the presence of masker stimuli. Histograms show the median delay shifts between target and mixture response in collicular (**A**) and cortical (**B**) units. Respectively, negative and positive values indicate delay shifts towards longer and shorter delays in response to the mixture condition. (**C**) Boxplots summarize the median delay shifts in the inferior colliculus (IC) and auditory cortex (AC). (**D**,**E**) Histograms show the target detection, indicated by cumulative d′ values, at neuronal level in the mixture situation in the IC (**D**) and AC (**E**). (**F**) Boxplots summarize the cumulative d′ values calculated for the IC and AC.

To quantify the effects of the masker on distance processing, we compared the “median echo-delays” in response to the target and the mixture. Based on the spike times, each spike was associated to a particular echo-delay of the target. The median of those delays represent the “median delay” of the unit. In response to the single animal mixture, the median delay did not change for 28.6% and 33% of the collicular and cortical units, respectively (black bars in Fig. 3A and B at Δ median delay = 0). In the AC, the colony masker evoked a stronger median delay shift than the single animal masker (Wilcoxon signed rank test: p < 10^−5^; Fig. 3C). Similar results were obtained by considering the “best delay”, represented by the echo delay evoking the peak in the PSTH, instead of the median delay (Fig. S4C–E). Note that the median delay usually shifted towards longer delays in the mixture situation. The median delay was more robust and shifted less in the IC than in the AC (Kruskal-Wallis test and Dunn’s multiple comparison post-hoc test: p < 10^−5^; Fig. 3C).

To quantify whether the target is encoded in the responses to the mixtures, we calculated d′ values with equations from the signal detection theory for each unit (IC Fig. 3D; AC Fig. 3E^42,43^). D’ values represent differences of the spike rate obtained in response to the masker and the mixture. High d′ values either arise from a response to the target or from a response caused by an integration of masker and target. The time course of d′ values was obtained by calculating d′ values for each call-echo element of the target (for details see methods and Fig. S3). To exclude spike rate differences that putatively come from integration of masker and target, we considered only d′ values from call-echo elements that evoke a spike rate of at least 50% of the maximum spike rate in response to the target. The sum of these d′ values represent the cumulative d’ for each unit. High cumulative d′ values indicate strong responses to the target in the mixture condition. The data showed that cumulative d′ values were higher for the single animal than for the colony masker (Wilcoxon signed rank test: p < 10^−5^; Fig. 3F) and they were also higher in the AC than in the IC (Kruskal-Wallis test and Dunn’s multiple comparison post-hoc test: p < 10^−5^; Fig. 3F). We got similar results when considering all bins of the PSTH for calculating cumulative d′ values (Fig. S4F–H). To summarize, our data shows that there exist different strategies for information representation in noisy environments along the colliculo-cortical axis. Masker induced median delay shifts are weaker in the IC than in the AC. At the same time, target representation, as calculated by d′ values, is higher in the AC than in the IC.

### Masker effect on cortical target-distance map

**Figure 4:**
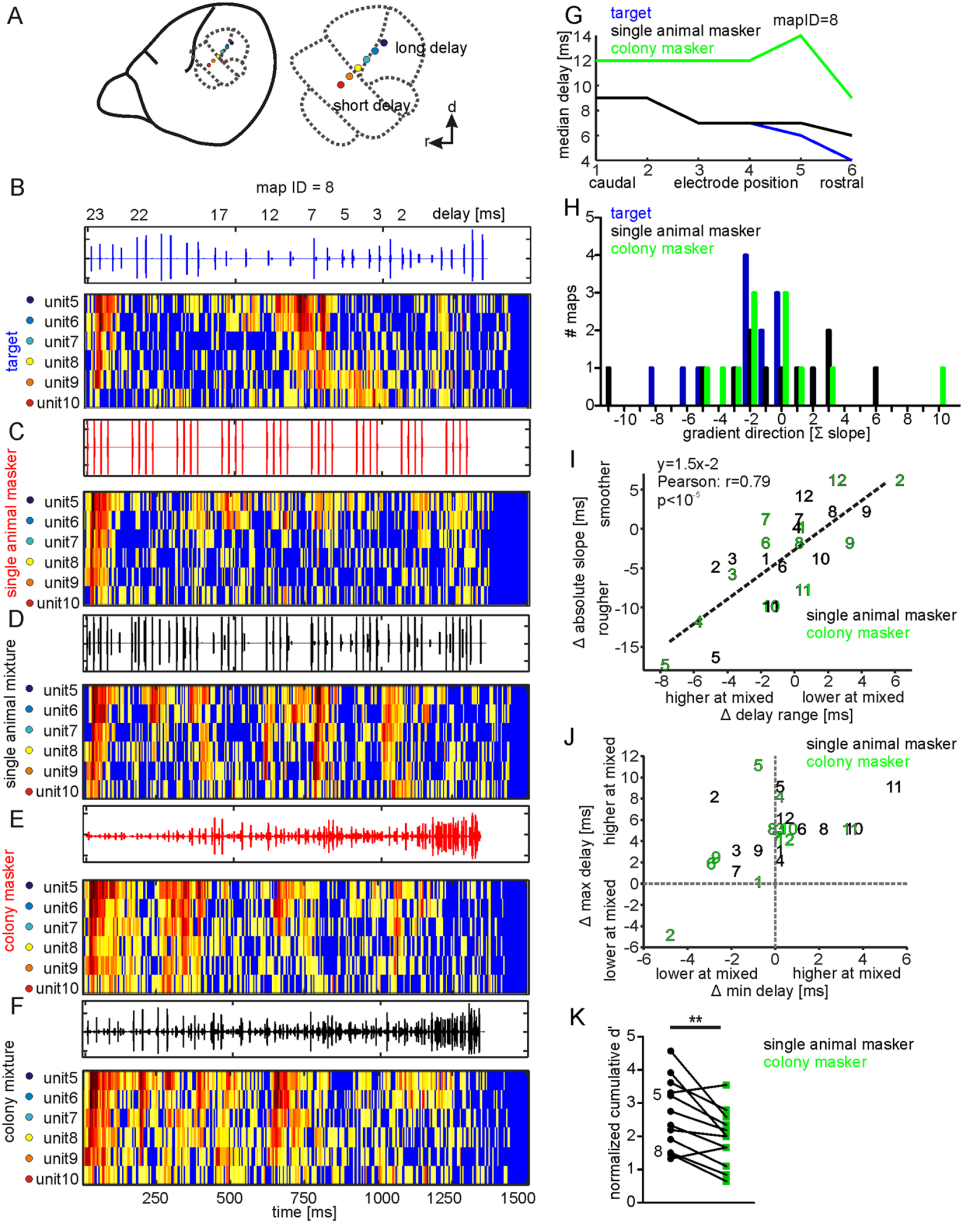
Robustness of chronotopy in the presence of masker stimuli. (**A**) Schematic lateral view on *C. perspicillata*’s brain and a magnification of the auditory cortex (dashed lines). (**B**–**F**) Color-maps represent neuronal activity (binsize = 5 ms) from a cortical map in response to the target (**B**), single animal masker (**C**), single animal mixture (**D**), colony masker (**E**), and colony mixture (**F**) condition. Each row represents a unit. Colored dots indicate electrode positions corresponding to the color code from (**A**). (**G**) Median delays in response to the target (blue), single animal mixture (black), and colony mixture (green) are plotted against the electrode positions for the example map. (**H**) Histogram shows the gradient direction of the maps. Negative values indicate a decrease from long to short delays along the caudo-rostral extent and vice versa. (**I**) For each map, the absolute median delay slopes and the delay range that the map covers were subtracted from each other and plotted for each map. Each number represents a map identifier (ID). The linear regression shows that the map roughness partially depends on the delay range. (**J**) The change in maximum delay is plotted against the change in minimum delay for each map and mixture condition. (**K**) Cumulative map d′ values are plotted for both mixture situations. The map-IDs of the example maps from (**A**–**F**) and Fig. S4 are indicated adjacent to the black data points.

In *C. perspicillata*’s AC, delay tuned neurons are topographically organized along the rostro-caudal axis^11,44^. Positioning multi-electrodes along the topographic gradient allows simultaneous characterization of long and short delay tuned neurons at caudal and rostral positions, respectively^26,45^, (Fig. 4A). We analyzed neuronal tuning in twelve cortical maps, eight from the left and five from the right cortex. Response patterns from two representative cortical maps are shown in Figs 4B–F and S5A–E. The topography was more distinct in response to the target (Figs 4B and S5A) than to the single animal (Figs 4D and S5C) and the colony mixture (Figs 4F and S5E).

The median delays plotted against the electrode position give an overview of the topographic changes induced by the masker (Figs 4G and S5F). In the presence of the colony masker, the topographic maps shifted towards longer delays than in the absence of the masker. Changes in neuronal tuning indicated by changes in delay tuning, response strengths at best delay, and cumulative d’ were independent of the electrode position (Fig. S5G–J).

The direction of the topographic gradient can be read out by the sum of the median delay slopes (Σ slopes; Fig. 4H). Negative Σ slopes signal a decrease of median delay from caudal to rostral and vice versa. In response to the target, the median delays decrease from caudal to rostral 9 out of 12 maps (target maps). Median delays from the three remaining target maps did not change along the rostro-caudal axis. Five single animal and three colony mixture maps, changed their direction of the gradient and had positive Σ slopes.

The smoothness of a topographic map is represented by the absolute slopes between adjacent electrodes. Abrupt delay shifts result in high absolute slopes, or rough topographic maps. Subtle delay shifts result in low absolute slopes, or smooth topographic maps. To identify whether mixture maps were rougher or smoother than target maps, the sum of the absolute slopes (Σ abs slopes) in response to the target was subtracted from Σ abs slopes in response to the mixture (Fig. 4I). Positive values, along the y-axis, indicate a smoother map in response to the mixture than to the target and vice versa. Map roughness increased in 58% (black numbers in Fig. 4I) and 67% (green numbers in Fig. 4I) of the maps in the single animal and colony mixture, respectively. Rougher maps are partially explained by an increase in the delay range that the map covers. The latter is indicated by a linear correlation between the delay range and the absolute slope (Pearson: r = 0.79, p < 10^−5^, Fig. 4I). In most cases, an increase of the delay range is a result of increasing the maximum delay instead of a decrease in the minimum delay (Fig. 4J). Cumulative d′ values were calculated and plotted for each map (Fig. 4K). The d′ values for the maps were significantly higher in the single animal masker than in the colony masker, indicating a higher target detection ability at the map level in the single animal mixture condition (Paired t-test: p = 0.005).

## Discussion

Many JARs have been reported in different species^3,7–9^. Generally spoken, acoustic signals are modified by the animals to make them distinct from the maskers. The validity of the JARs with respect to a putative facilitation of neuronal processing of targets remains largely unexplored. In the present study, *C. perspicillata* shows a JAR that is based on controlling the time points of call emission. Such JARs have been discussed in different animals, like crickets^46^, frogs^47,48^, electric fish^49^, birds^50,51^, bats^29,39,52,53^, and monkeys^54^. In response to the maskers, *C. perspicillata* emits tight groups of calls. The fact that 60% of the calls were emitted in groups, independent from the presence of the masker, shows the importance of emitting call groups during echolocation. In bats, the emission of call groups has been associated with a high complexity of orientation tasks^30,33–36,39,40^. Behavioral results in the frugivorous bat *Phyllostomus discolor* also show an increase of call grouping in the presence of maskers^29^, corresponding to the present results from *C. perspicillata*.

**Figure 5:**
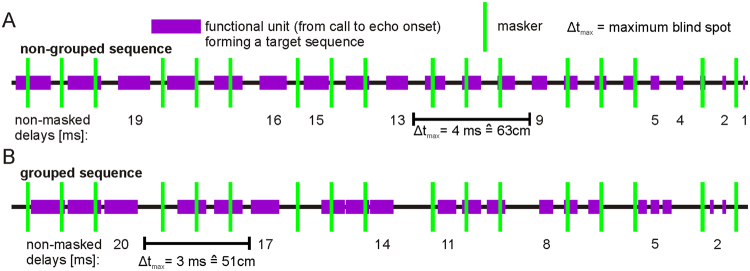
Grouping calls makes distance processing more robust against maskers. Two schematic echolocation/target sequences are presented in violet. Each violet bar represents a functional unit spanning the time from call to echo onset. Both sequences contain 21 functional units, but they have different time structures. In (**A**) the calls are equally distributed (non-grouped sequence) and the encoded echo delays range from 21 to 1 ms in 1 ms steps. In (**B**) the calls are grouped into triplets where each functional unit of a triplet encodes a similar delay (< ± 1 ms). The delay information encoded by a functional unit gets masked if a masker occurs during the functional unit. Non-masked delays are indicated below each sequence. Δt_max_ represents the maximum blind spot that the bat would perceive in case of the masker presence.

How could call grouping facilitate echolocation in the presence of maskers? For illustration, we consider the detection of two different target sequences (Fig. 5). Echolocation calls from the first target are equally distributed (non-grouped sequence; Fig. 5A) and the calls of the second target are grouped into triplets (grouped sequence; Fig. 5B). According to a median CI of 22 ms (red boxplot in Fig. 1F) and an average flight speed of 2.5 m/s^28^, a triplet carries distance information from ±11 cm. Thus, the call-echo elements, or functional units of a triplet, encode echo delays in the range of ±1 ms. If no functional unit gets masked, then the non-grouped sequence conveys higher spatial resolution (1 ms) than the grouped sequence (3 ms). However, if functional units get masked then two functional units from a triplet of the grouped sequence could get masked without losing much delay information. In contrast, the loss of delay information might be more dramatic if functional units get masked in the non-grouped sequence. Accordingly, the maximum distance that the bat must cover without delay information (maximum blind spot) is longer in the non-grouped than in the grouped sequence (in our example, 63 cm for the non-grouped and 51 cm for the grouped sequence). In summary, grouping the calls more tightly increases the discontinuity of echo information but it could increase the continuity of preserved delays (compare the continuity of preserved delays in (A) and (B)).

At the neuronal level, call groups and maskers increase the acoustic rate which could potentiate neuronal suppression^26,45^. Neuronal suppression is usually weaker at subcortical than at cortical areas^21,25,55^. This may explain, why the maskers tend to induce stronger delay tuning shifts in the AC than in the IC. In the AC, delay tuning was usually shifted towards longer delays which could be based on two aspects: i) In noisy environments, the cortex is biased to process echo information from distant objects, like conspecifics. The latter is especially important for tracking flight trajectories of conspecifics for avoiding collisions. ii) The risk of interference is highest at long delays because the functional units span a longer duration than the ones at short delays (Fig. 5). Thus, delay tuning shifts towards long delays could reflect jamming effects.

The target representation in response to the mixture was computed by d′ values that have been used in previous studies^42,43^. Corresponding to the high d′ values in the AC, target detection in the mixture situation was higher in the AC than in the IC. This result could be explained by the differences of suppression strengths that affect the neuronal selectivity in both brain areas^56^. In contrast to collicular neurons that usually respond to each acoustic event of an echolocation sequence^25^, cortical neurons respond selectively to certain acoustic events^26^. The increased selectivity makes the AC less sensitive to the maskers which results in higher d′ values than in the IC (compare responses to colony masker in Fig. 2D). The fact that the increase of neuronal selectivity along the ascending auditory pathway could be correlated with an improvement of target detection in a noisy situation is supported by findings in frogs. The ability of target detection^57^ and the neuronal selectivity^4,58^ increases from the auditory nerve to the IC of frogs.

Note that during the neuronal recordings, the animals were passively listening to echolocation sequences. Attentional effects, binaural effects, directionality of the stimuli and of hearing could further facilitate signal processing under noisy conditions^4,31,56,59,60^. A rhythmic sampling of the environment that is induced by emitting call groups might be beneficial, because the bats could attend and predict the temporal pattern of echo arrival. Neuronal activity usually oscillates over time which is often associated with attentional phenomena^61,62^. If the rhythmic sampling of the environment occurs in phase with ongoing brain oscillations, then it might happen that the animal’s attention is high at the expected time points of echo perception^61^. The latter is conceptualized by the “temporal binding hypothesis”^62,63^ which states that stimulus features coming from one source like a single object are combined through a synchronized neuronal activity. Stimulus features that do not belong together are not temporally bound or synchronously processed. Thus, although interference signals might evoke neuronal activity, this activity is likely not associated with the ethologically relevant stimuli when the information is not synchronously processed.

In summary, the present results indicate that in noisy environments, *C. perspicillata* increases the discontinuity of echo information by emitting tightly grouped calls. Each functional unit of the group encodes a similar echo delay which makes some echoes expendable for the bat. Erroneously encoded distance information could get updated at neuronal level. At the neuronal level, moderate delay tuning shifts in the IC and high d′ values in the AC indicate that target information is still encoded in the presence of the masker. Thus, the JARs, found in *C. perspicillata*, could potentially facilitate neuronal processing of natural and ethologically relevant stimuli.

## Methods

### Animals

Behavioral experiments were conducted in eight (1 female, 7 males) bats of the species *Carollia perspicillata* and the electrophysiological experiments were conducted in 15 female bats (9 for auditory cortex and 6 for inferior colliculus). All bats were bred in a colony at the Institute for Cell Biology and Neuroscience (Frankfurt University). The experiments comply with all current German laws on animal experimentation and they are in accordance with the Declaration of Helsinki. All experimental protocols were approved by the Regierungspräsidium Darmstadt (experimental permit # F104/57).

### Behavior

Behavioral experiments were performed in a wooden flight chamber (Movie 1–3; Fig. 1A; length: 4 m; width: 1.4 m; height: 2 m). A wall, made from foam, separated the room into two sides, each side measuring 2.5 m × 0.65 m × 2 m. A landing platform (20 × 20 cm), made of metal mesh, was positioned in one of the walls in each side of the room. Behind each metal mesh, one speaker (Neo CD 1.0 Ribbon Tweeter; Fountek Eelctronics, China) and one ultrasound sensitive microphone (Avisoft Bioacoustics, Germany) were installed. The speaker could produce sounds above 90 dB SPL in the frequency range from 5–130 kHz (calibration curve obtained with a ¼-in. microphone (model 4135; Brüel & Kjaer) + a custom-made microphone amplifier). In the calibration curve, SPL decreased at a rate of 0.13 dB/kHz as sound frequency increased from 5 to 130 kHz. The microphones had a sensitivity of 50 mV/Pa and an input-referred self-noise level of 18 dB SPL. Their frequency response curves were flat (+/−3 dB, as specified by the manufacturer) in the range from 30–130 kHz. Each microphone was connected to a sound acquisition system (one microphone to an UltraSoundGate 116 Hm mobile recording interface and the second microphone to an UltraSoundGate 116 Hb mobile recording interface, + Recorder Software, Avisoft Bioacoustics, Germany) for sound digitalization at 333 kHz (16 bit precision).

During the training trials, call emissions of hand released bats were acoustically recorded with the microphones. The flight behavior was monitored with a webcam (500 SX, Manhattan, USA) placed above the starting point and with a frame rate of 30 Hz. During test trials, one speaker produced playback stimuli resulting in a potentially masking side. The speaker of the contralateral side remained silent (non-masking side). Masking and non-masking sides were randomly selected. The playback stimuli represented repetitions of a representative biosonar call that was recorded during a training trial from the tested animal. The biosonar call was repeated in groups of five (one animal), ten (four animals) or twenty calls (three animals) with within group call intervals of 15 ms and in between group intervals of 35 ms. The within group call intervals of 15 ms lies in the range of the minimum pulse intervals produced by *C. perspicillata*^28^. Behavioral data from bats that were stimulated with call groups consisting of five, ten, or twenty calls were not different and therefore were grouped together for data analysis. Acoustic stimuli were generated with a sampling rate of 384 kHz with an Exasound E18 sound card (ExaSound Audio Design, Canada), and sent to an audio amplifier (Rotel power amplifier, RB-850, USA). The stimuli were played with a sound pressure level of 80–90 dB re 20 µPascal (dB SPL).

The call emissions were recorded by the microphones and a segment of two seconds was analyzed from each trial. The segment was chosen such that the echolocation calls with the highest amplitude during the trial were considered. This reduced the risk of missing echolocation calls whose amplitude are too low to be recorded. The analysis segments from test trials were chosen from recordings conducted while the animals flew at the masking side. In total, 48 segments from eight animals were analyzed. Twenty-four segments (3 per animal) were recorded during the training and another 24 were recorded during the test trials. Call emission patterns during test and training trials were compared pairwise, meaning that three pairs of “test” and “training” trials were compared for each animal.

For data analysis, the time points of call emissions were manually tagged in the software Avisoft SAS Lab Pro (Avisoft Bioacoustics, Germany). These time points were later used for the remaining analysis done in Matlab 2014 (MathWorks, USA). Call groups were defined according to the criterions formulated by^30^. First an “island criterion” defines temporally isolated call groups. The island criterion is fulfilled when the preceding and following call intervals of a call group are 20% longer than the call intervals within call groups. If the island criterion is fulfilled a second criterion, the so called “stability criterion”, defines the size of the call groups indicated by the number of calls belonging to a group. The stability criterion is fulfilled if the call intervals within call groups are stable with a 5% tolerance. Note that doublets, i.e. call groups containing two calls, can only be defined according to the island criterion. For determining triplets, quartets, quintets or sextets, both criteria had to be fulfilled. The behavioral analysis considered doublets to sextets, because sextets represent the longest call groups reported in *C. perspicillata*^27^.

### Stimuli for electrophysiology

To study neuronal responses, we used two types of “maskers” and one “target” stimuli. The target was an echolocation sequence recorded from a pendulum flight simulating an approach flight^26^. In a previous article, we had already shown that the target evoked reliable neuronal responses in high-frequency tuned neurons of the IC and AC of *C. perspicillata*^25,26^. Note that in the target, the echo delays decreased from 23 ms at the first, to 1 ms at the last call-echo element. The recording and preparation of the echolocation sequence was explained in detail in a recent study^26^. Briefly, the acoustic signals were recorded using a pendulum paradigm^64^, in which the bat was positioned in a pendulum mass and it was swung towards an acrylic glass wall^26^. During the swing, the bat emitted sequences of echolocation calls that were recorded, together with their echoes, with the aid of an ultrasound sensitive microphone attached to the pendulum (Avisoft Bioacoustics, Germany). The SPL of the acoustic events of the target echolocation sequence varied between 36–77 dB SPL.

As masker stimuli three different sound sequences were used. Two of them were defined as “single animal masker” and the remaining one was described as “colony masker”. The single animal maskers consisted of echolocation calls coming from one individual. One of the single animal maskers contained 33 calls that were recorded with the pendulum paradigm. Echoes were manually deleted from the sequence. In other words, except the missing echoes, the single animal masker was natural in the sense of spectral, temporal, and intensity parameters. The SPL of the calls varied between 62 and 82 dB (median = 71 dB SPL). The second single animal masker was defined as semi-natural because the level, spectrum and temporal properties of the natural echolocation calls that composed this masker were stable throughout the stimulation. The echolocation call, used as building block for the second single-animal masker was repetitively presented to the animals in form of quartets. The CIs within call groups was 23 ms and 83 ms between groups. The single animal masker simulates an acoustic environment where two bats echolocate close to each other. Data obtained with both single animal maskers were comparable, and therefore they were grouped together for analysis.

In comparison to the single animal masker, the colony masker contained more acoustic events, including calls, echoes, and communication calls from a colony of *C. perspicillata* with 150 animals. The colony masker was recorded with an ultrasound sensitive microphone that was held for 30 seconds inside of the colony room of the facility. A segment of 1.34 seconds of the recording was used as colony masker. The masker contained more than 200 acoustic events that partially overlapped in time and the SPL of the acoustic events ranged from 43 to 81 dB (median = 63 dB SPL). Due to temporal overlap, it was impossible to measure the exact number of acoustic events in the colony masker. The colony masker reflects a natural acoustic environment that *C. perspicillata* has to face in the roosts. The “single animal mixture” and the “colony mixture” represent stimuli that were obtained by adding the target stimulus to the single animal and colony maskers, respectively. Stimuli were presented fifteen times.

### Electrophysiological recordings

Electrophysiological recordings took place in a sound-proofed and electrically-shielded chamber. Recordings from the IC were focused on the central nucleus of the left IC whose position was determined based on the tonotopic arrangements of the recorded units. Neuronal signals from the AC were recorded in the left (n = 8) and right (n = 5) hemispheres. For the surgery, the bats were anaesthetized with a subcutaneous injection of a mixture of ketamine (10 mg/kg Ketavet, Pharmacia GmbH, Germany) and xylazine (38 mg/kg Rompun, Bayer Vital GmbH, Germany). A longitudinal midline incision was made through the skin overlying the skull. Muscle tissue, covering dorsal and temporal parts of the skull, was removed. For cortical recordings, a craniotomy was made over the high frequency area of the brain, to gain access to auditory neurons. For subcortical recordings, a craniotomy above the sulcus separating the cerebrum and cerebellum, gave access to the IC. For the fixation of the bat’s head, a custom-made metal rod (1 cm length, 0.1 cm diameter) was glued onto the skull using dental cement (Paladur, Heraeus Kulzer GmbH, Germany). Each bat was used for chronical recording sessions that lasted several hours over a period of two weeks. At the day of recording, the animals were lightly anaesthetized with a small dose (0.03 mL) of ketamine/xylazine mixture diluted in sodium chloride. This small dose allowed us to place the animal in the recording setup and to position the electrodes either in the AC or IC. Neural data acquisition started as soon as the animals woke up from anesthesia. That the bats had woken up was assessed by spontaneous and auditory evoked movements of the pinna, mouth, and nose-leaf. These movements were first visible about one to two hours after the initial dose of anesthesia.

For cortical recordings, two electrode types were used. (i) Commercially available micro-electrode arrays with 16 recording electrodes organized in 2 × 8 (MicroTargets for Life Science, USA). A reference electrode with an impedance of 10 kΩ was placed adjacent to the recording electrodes. Reference and recording electrodes were made out of tungsten whereas a silver wire placed on the cortical surface of the frontal cortex was used as ground. Each recording electrode had an impedance of 2 MΩ (as reported by the manufacturer). The arrays had an electrode and row spacing of 250 µm. (ii) Custom-built glass electrode arrays of up to 8 channels organized in a single row. Glass electrodes (resistance 1–10 MΩ when filled with 3 mol/L KCl) were pulled from borosilicate capillaries (GB120F-10, Science Products, Germany) with a Flaming/Brown horizontal puller (P97, Sutter, USA) and they were glued together in a fan-shape pattern, ensuring an electrode tip spacing of 250 µm. For IC recordings, single glass electrodes were used with the same specifications as for the glass electrodes of the custom-built glass electrode arrays.

A wireless multichannel recording system (Multi Channel Systems MCS GmbH, Germany) was used for data acquisition at a sampling rate of 20 kHz per channel and 16 bit precision. Action potentials were filtered using a 2^nd^ order Butterworth band-pass filter, with cutoffs between 300–3000 Hz.

### Spike-Data Analysis

Spike detection was based on spike amplitude relative to recording noise level. The spikes were sorted based on the first three principal components of the spike waveforms and they were clustered automatically using the “KlustaKwik” algorithm^65^. Only the cluster containing the largest amount of spikes was used for analysis.

Neuronal data from the AC comprised 72 spike-sorted single-units that were recorded from twelve cortical maps. Between three and twelve units were recorded simultaneously (median = 6). Twenty units were recorded with the commercially available micro electrode arrays from Microprobes and 52 units were recorded with the custom-made glass electrode arrays. Neuronal data from the IC comprised 49 spike-sorted single-units that were sensitive to high frequencies (>40 kHz).

Data analysis was based on post-stimulus time histograms (PSTHs) constructed with a binsize of 5 ms and 2 ms for cortical and collicular data, respectively. Different binsizes between collicular and cortical units were used because collicular neurons fire temporally more precise than cortical ones^19^. The initial 150 ms of the cortical response were not considered because of strong stimulus independent onset responses^26^. Delay tuning to the target stimulus was assessed by assigning each spike, according to its occurrence, to a specific echo delay of the echolocation sequence used as stimulus. The assignment of each spike to a specific delay allowed us to reconstruct delay tuning curves. The best delay is defined based on the call-echo element eliciting the strongest response i.e. the largest number of spikes. The median delay was calculated by measuring the median time of occurrence of the evoked spikes. The median time point was then assigned to the preceding call-echo element whose echo delay represents the median delay. In comparison to the best delay, which reflects the maximum response only, the median delay calculation considers each elicited spike. A response to certain echo-delays was considered to occur if the neuronal response to the target sequence was at least as strong as 50% of the maximum response observed (i.e. the response strength at the best delay).

For quantifying the preservation of the response to the target in the mixture situation (target detection), we calculated unit specific cumulative d′ values (Fig. S3). First we determined median d′ values from the response window to each call-echo element of the target.

1 $${d^{\prime} }_{i}^{2}=\frac{{(mi{x}_{i}-{m}_{i})}^{2}}{0.5\,[\sigma {(mi{x}_{i})}^{2}+\sigma {({m}_{i})}^{2}]\,}$$

In the equation (1), *mix*_*i*_ and *m*_*i*_ represent the median spike rate in response to the call-echo element in the mixture and masker situation, respectively. Thus, high spike rate differences between the response to the mixture and masker result in high d′ values. These differences are due to the presence of the target in the mixture and may either arise from responses to the target or from an integration of the target and masker stimulus. A neuronal response arising from stimulus integration represents a neuronal correlate of jamming. To determine the target detection in the mixture situation it is necessary to exclude high d′ values arising from jamming. Therefore, we calculated a cumulative d’ for each unit by considering only d′ values from call-echo elements that elicited a neuronal response in response to the target stimulus *i*. Cumulative d′ values were calculated according to the following equation (2):

2 $$cumulative\,d^{\prime} =\sqrt{{\sum }_{i}{d^{\prime} }_{i}^{2}}$$

The higher the neuron’s cumulative d’, the higher is its target detection in the mixture situation. The quantification of target detection based on d’ have been successfully performed in previous studies^42,43^. Data analysis was done in Matlab 2014 and statistics in GraphPad Prism 5 (GraphPad Software, USA; *p < 0.05; **p < 0.01; ***p < 0.0001).

## Electronic supplementary material

Movie 1

Movie 2

Movie 3

Supplementary_material
