# Supplementary material for: Robustness of cortical and subcortical processing in the presence of natural masking sounds
Source: Sci Rep. 2018 May 1;8:6863. doi: 10.1038/s41598-018-25241-x (PMC5931562; doi:10.1038/s41598-018-25241-x)
Supplement: Supplementary file 4 — Supplementary_material [file 41598_2018_25241_MOESM4_ESM.pdf]

***Title*: Robustness of cortical and subcortical processing in the presence of natural masking sounds**

***Authors*:** M. Jerome Beetz (*)[1,2], Francisco García-Rosales[1], Manfred Kössl[1], Julio C. Hechavarría[1]

***Affiliations*:**

[1]Institute for Cell Biology and Neuroscience, Goethe-University, 60438, Frankfurt/M., Germany

[2]Department of Behavioral Physiology and Sociobiology, Biozentrum, University of Würzburg, Am Hubland, Würzburg 97074, Germany

* Corresponding author

***Mailing address*:**

M. Jerome Beetz

Email: beetzjerome@gmail.com

Lehrstuhl für Zoologie II, Am Hubland, 97074 Würzburg, Germany

***Short Title:* Robust neuronal processing in noisy environments**

Supplementary material

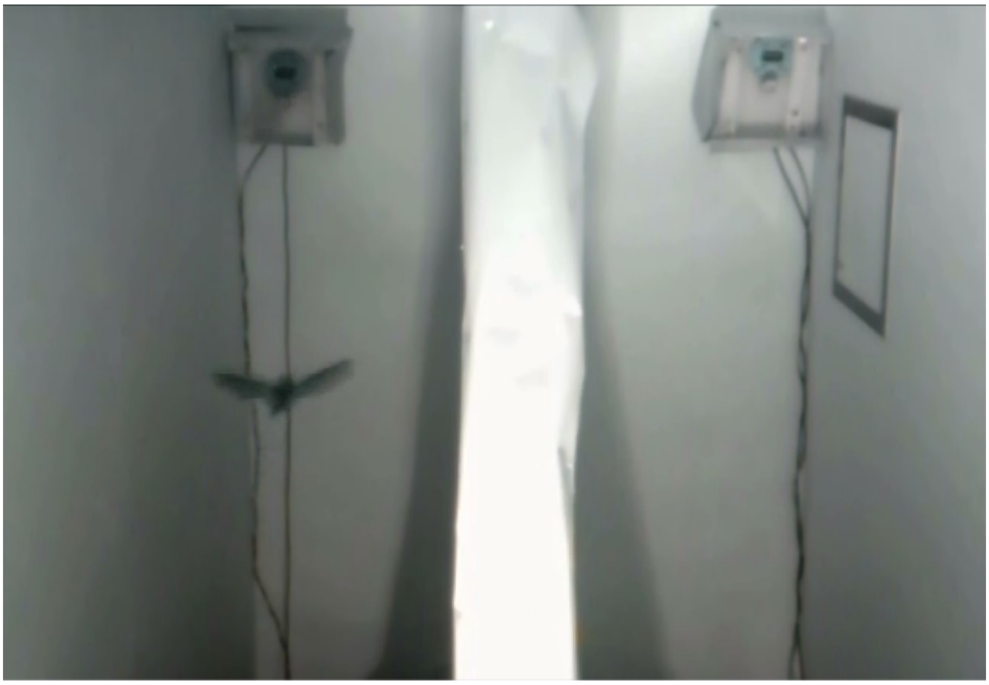

Movie S1 Exemplarily training trial. The bat was hand released from a starting point and it landed on the left platform.

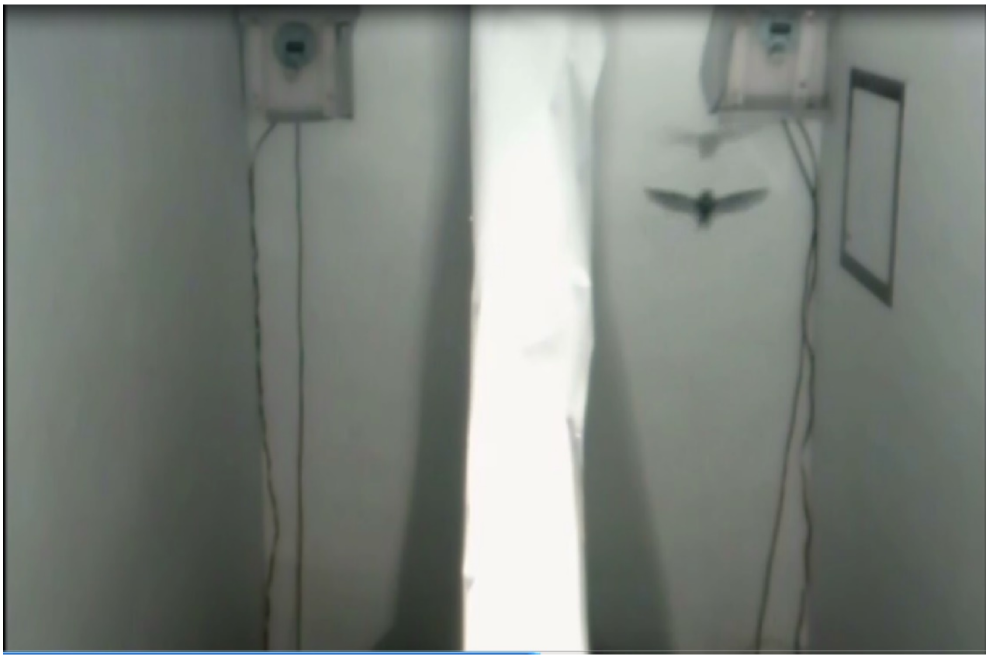

Movie S2 One test trial showing that the bat avoids to land on the platform at the masking side. Masking stimuli were presented from the left side.

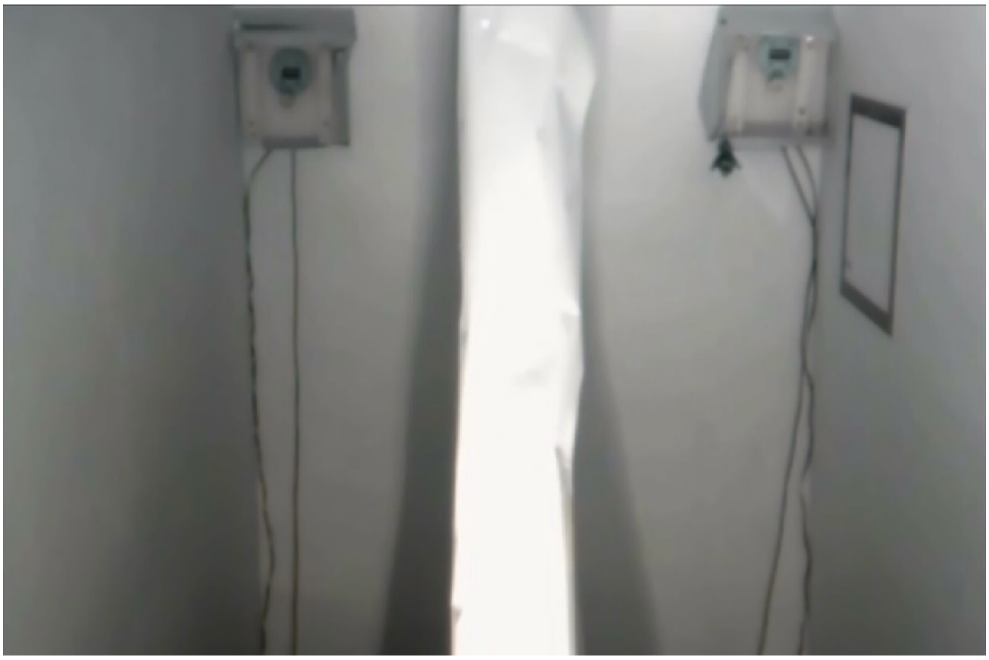

Movie S3 One test trial showing that the bat could still land on the platform at the masking side. Masking stimuli were presented from the right side.

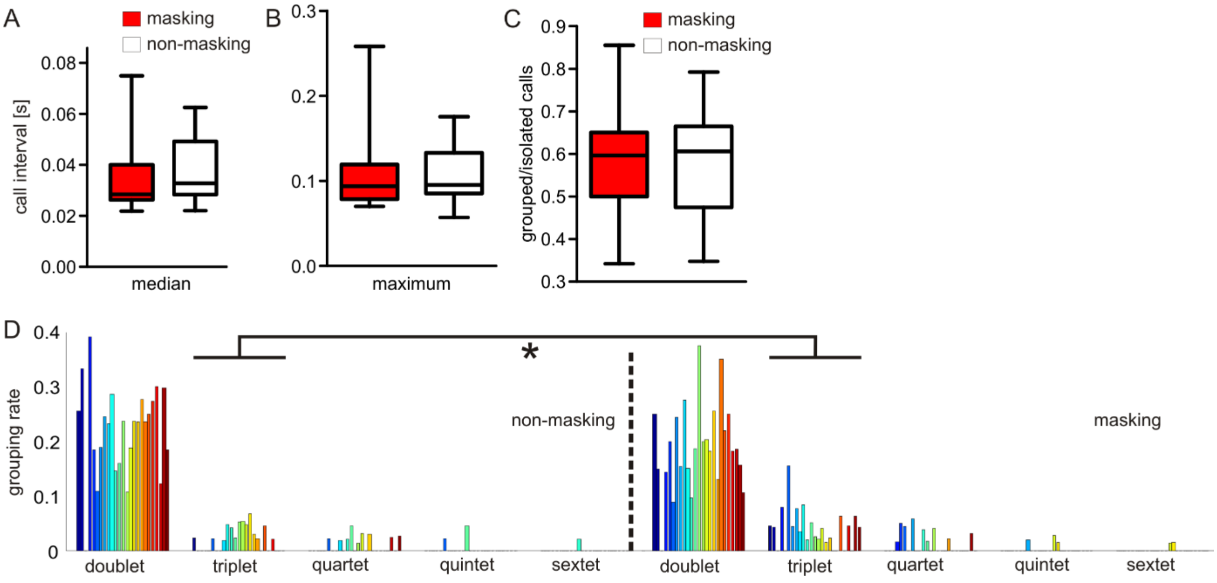

Figure S1 Behavioral adaptations of *C. perspicillata* when echolocating in noisy environments. (A) Median and (B) maximum Call intervals were not affected by playback stimuli. (C) Relative amount of calls emitted in groups did not differ significantly between masking and non-masking conditions. Note that most calls (about 60% in both conditions) were emitted in groups. (D) Histograms showing the relative amount of call groups consisting of two (doublet), three (triplet), four (quartet), five (quintet) and six (sextet) calls in the non-masking and masking condition. Each color represents one pair of trials

(24 pairs, 3 pairs each animals) of one animal. Under masking conditions more triplets were emitted than under non-masking conditions.

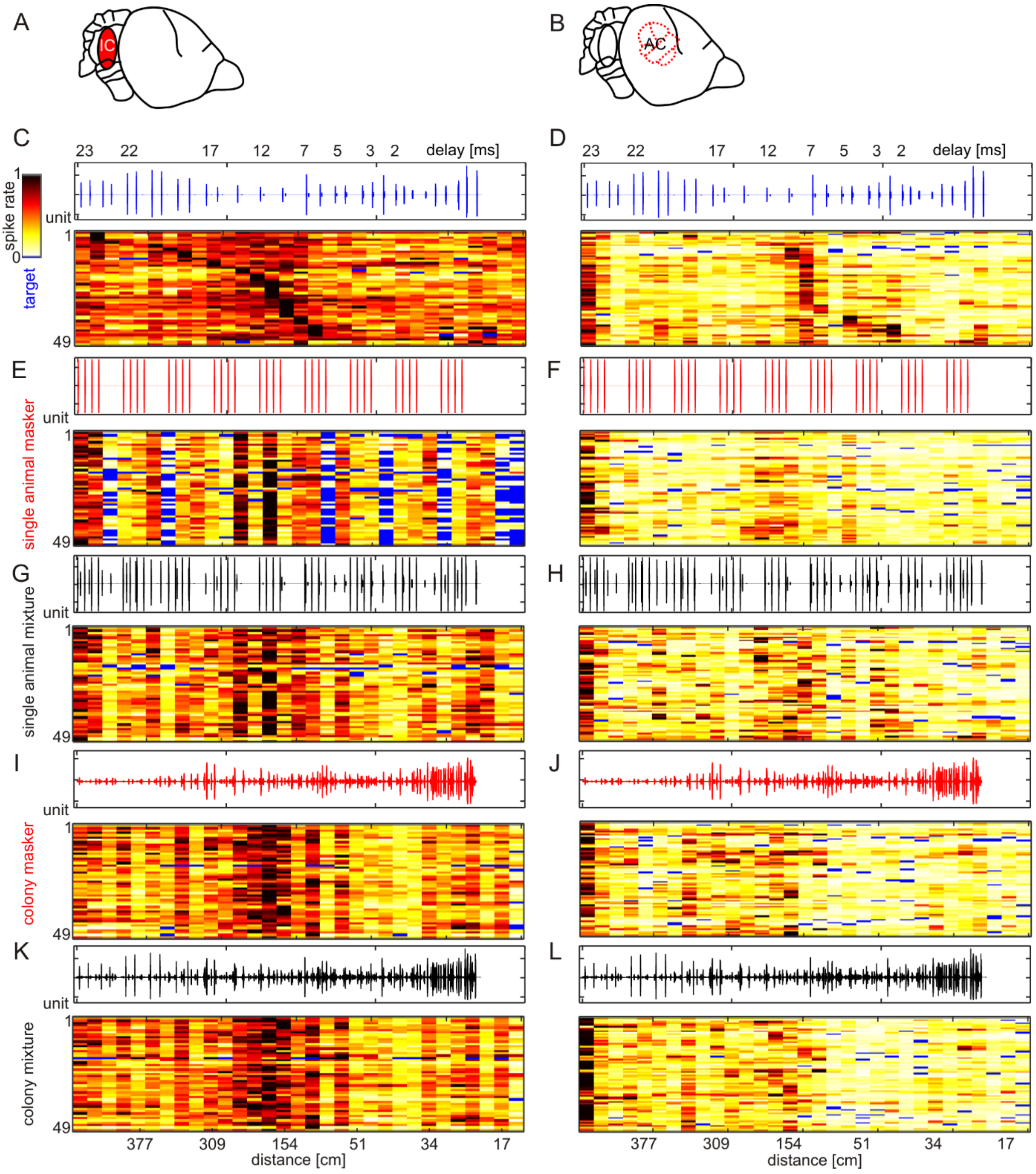

Figure S2 Neuronal responses of 49 collicular and 72 cortical units.

(A) Schematic lateral view on *C. perspicillata*'s brain. Recorded brain areas are highlighted (inferior colliculus (IC); auditory cortex (AC)). (B-F) Color-maps representing the neuronal activity (binsize according to duration of call-echo element) from 49 collicular (left column) and 72 cortical (right

column) units in response to the target (B), single animal masker (C), single animal mixture (D), colony masker (E), and colony mixture (F) condition. Each row represents a particular unit and the units are ordered according to their best delay. Spike rate was normalized to the maximum bin for each unit and stimulus condition.

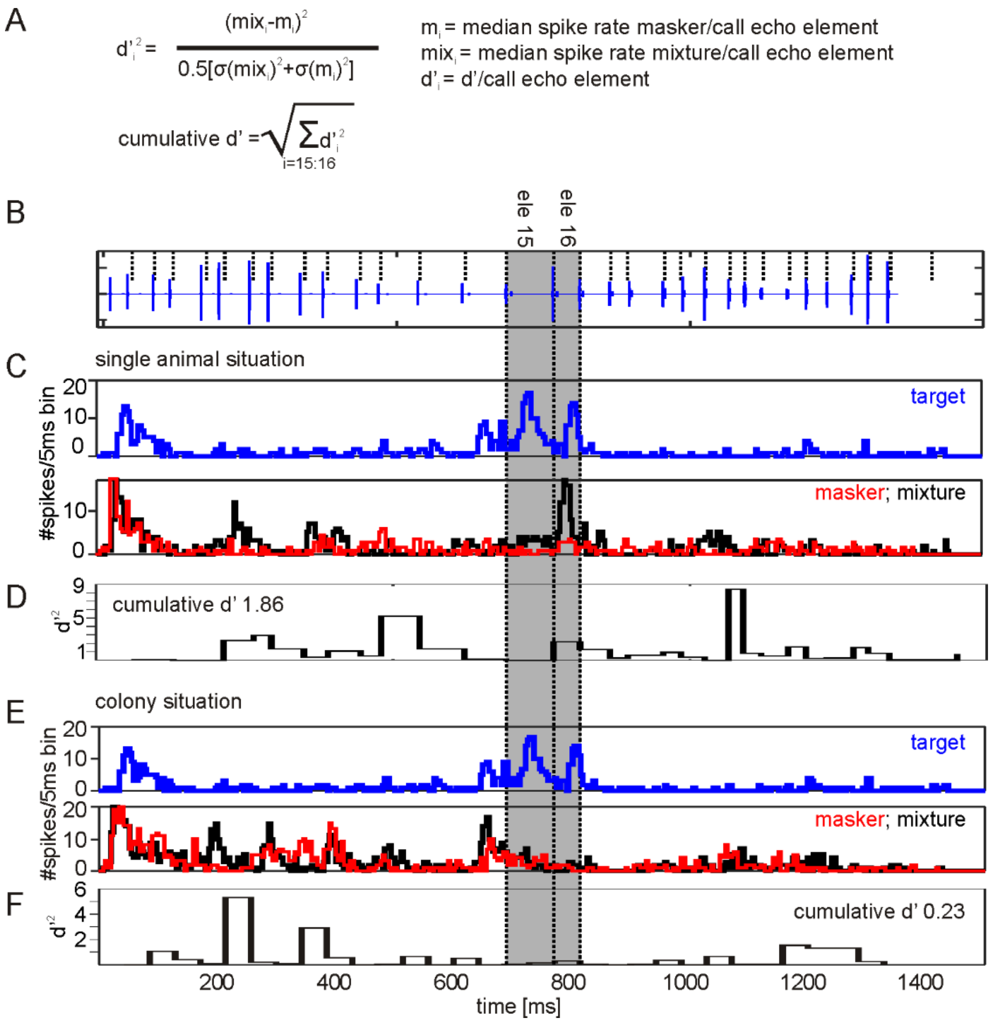

$$d'^2_i = \frac{(mix_i - m_i)^2}{0.5[\sigma(mix_i)^2 + \sigma(m_i)^2]}$$

$$\text{cumulative } d' = \sqrt{\sum_{i=15:16} d'^2_i}$$

Figure S3 D' calculation exemplified by a cortical unit.

(A) Equations used to calculate a cumulative d' for each neuron. (B) Oscillogram of the target. Dashed vertical grey lines indicate the time borders of the response windows for each call-echo element. (C) PSTHs calculated in response to the target (blue), single animal masker (red) and single animal mixture (black). (D) Histogram shows the d'^2 for each call-echo element of the example unit. Note that high spike rate differences between the red and the black PSTH results in high d'^2 values. For calculating a cumulative d' for the unit, the d' values from call-echo elements to which the neuron responded to (ele 15 and ele 16) were summed. (E, F) The same figures as in (C, D) but in response to the colony masker

and colony mixture. The response to the target was more preserved in the response pattern to the single animal mixture than to the colony mixture. The latter is evident when comparing the blue with the black PSTHs and it can be quantified by the cumulative d'.

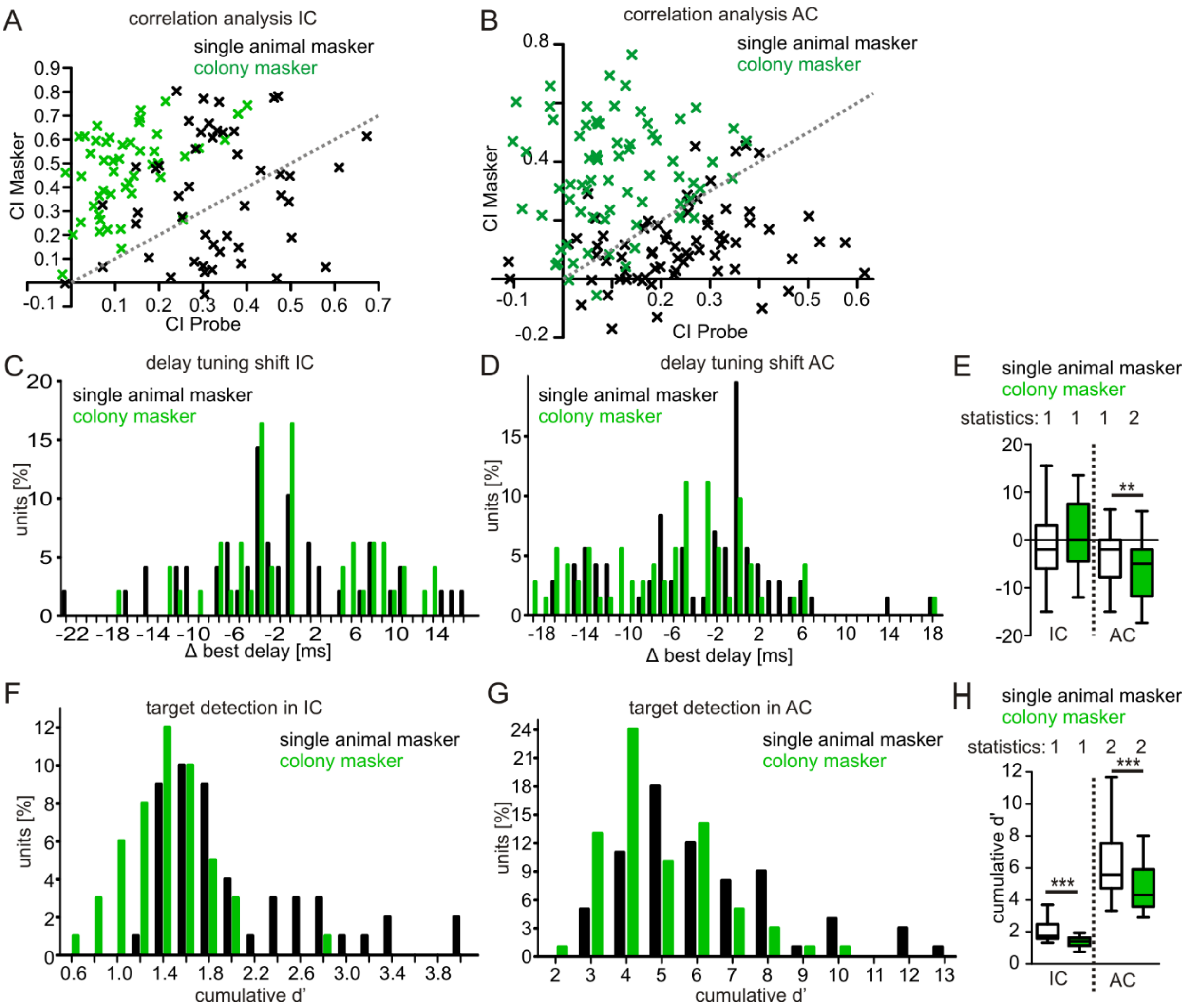

Figure S4 Robustness of neuronal processing in the presence of masker stimuli.

(A-B) Distribution of masker CIs are plotted against the target CIs from all collicular (A) and cortical (B) and units and both stimulus conditions. Values from single animal masker and mixture condition are plotted in black and the values from colony masker and mixture condition in green. (C, D) Histograms show the best delay shifts between target and mixture response in collicular (C) and cortical (D) units. Respectively, negative and positive values indicate delay shifts towards longer and shorter delays in response to the mixture condition. (E) Boxplots summarize the best delay shifts in the inferior colliculus (IC) and auditory cortex (AC). In the AC, the colony masker induced a higher best delay shift

than the single animal masker. (F, G) Histograms show the target detection, represented by cumulative d’ values, in the mixture situation in the IC (D) and AC (E). Note than in comparison to figure 4D and 4E all call-echo elements were considered to calculate the cumulative d’ values. (F) Boxplots summarize the signal detection of the target in the presence of the masker. The cumulative d’ and thus the neuronal signal detection were higher in the single animal than in the colony masker. Cumulative d’ were also higher in the AC than in the IC.

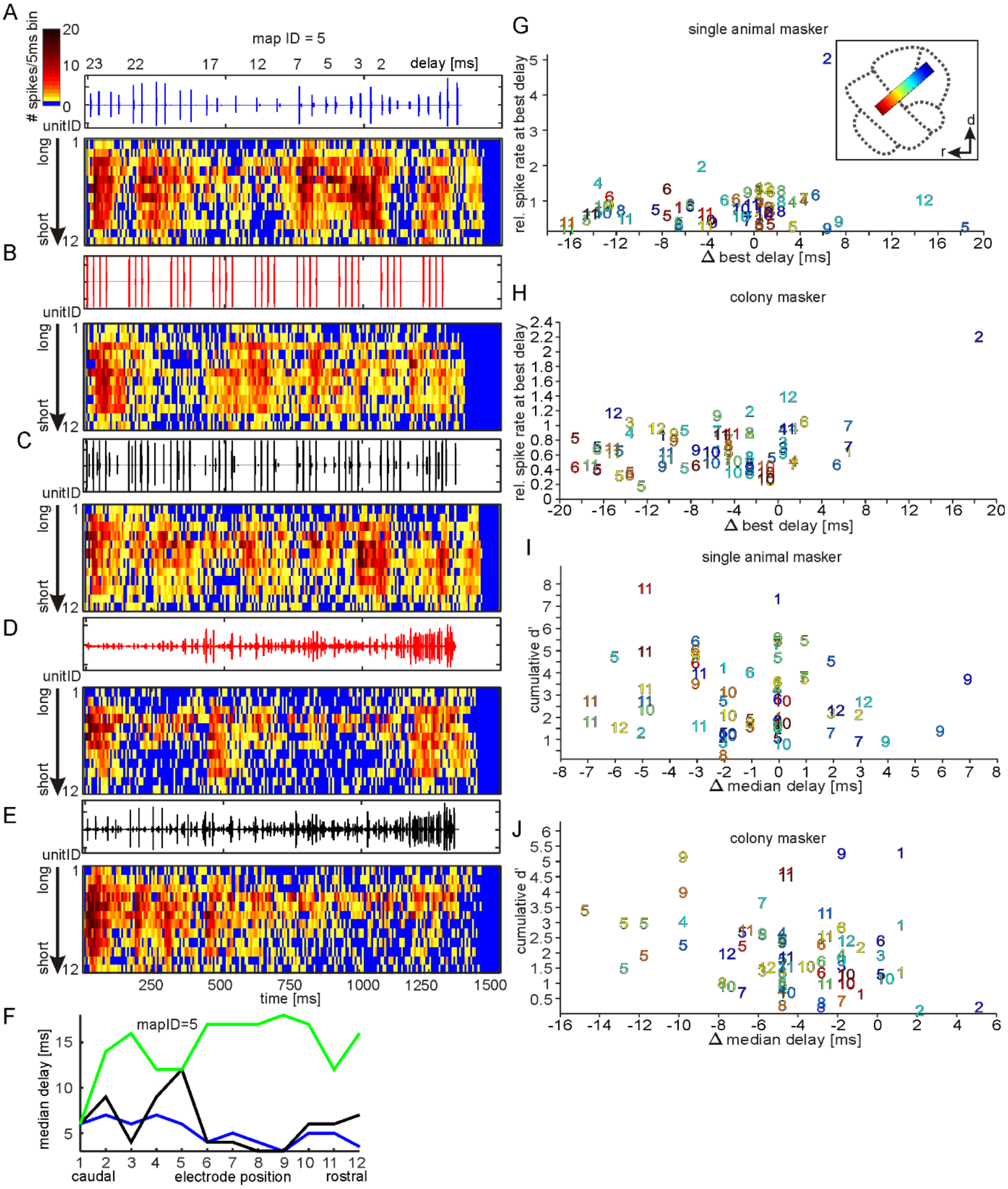

Figure S5 Robustness of chronotopy in the presence of masker stimuli.

(A-E) Color-maps representing neuronal activity (binsize = 5 ms) from a cortical map in response to the target (A), single animal masker (B), single animal mixture (C), colony masker (D), and colony mixture (E) condition. Each row represents a unit. (F) Median delays in response to the target (blue), single animal mixture (black), and colony mixture (green) are plotted against the electrode positions for the example map. (G-H) Best delay shifts between the responses to the target and mixture were plotted against the relative spike rate to the best delay of the target sequence. Data points from single animal masker are plotted in (G) and from the colony masker in (H). In total twelve cortical maps were investigated and the map ID to which the particular data point belongs to is coded by a number (ranging from 1-12). Electrode position is color coded as represented in the scheme in (G). (I-J) Median delay shifts between the responses to the target and mixture were plotted against the cumulative d'. Only call-echo elements to which the neuron was sensitive to in the target sequence was considered to calculate the cumulative d'. Data points from single animal masker are plotted in (I) and from the colony masker in (J).
